# The Role of Sports in the Subjective Psychological Well-Being of Hungarian Adult Population in Three Waves of the COVID-19 Pandemic

**DOI:** 10.3390/ijerph20010660

**Published:** 2022-12-30

**Authors:** Tamás Laczkó, Pongrác Ács, Kata Morvay-Sey, Bence Cselik, Miklós Stocker

**Affiliations:** 1 Institute of Health Insurance, Faculty of Health Sciences, University of Pécs, Vörösmarty u.3, 7621 Pécs, Hungary; 2 Physical Activity Research Team, Szentágothai Research Centre, University of Pécs, Ifjúság útja 20, 7624 Pécs, Hungary; 3 Institute of Physiotherapy and Sport Sciences, Faculty of Health Sciences, University of Pécs, Vörösmarty u.3, 7621 Pécs, Hungary; 4 Institute of Strategy and Management, Corvinus University of Budapest, Fővám tér 8, 1093 Budapest, Hungary

**Keywords:** subjective psychological well-being, WHO-5, sports, physical activity, COVID-19 pandemic

## Abstract

(1) Background: In this study, sport and subjective psychological well-being is investigated in three waves of the COVID-19 pandemic. (2) Methods: We have conducted three different representative sample surveys (n = 3600 altogether) on the Hungarian adult population and investigated the sample’s subjective psychological well-being with the WHO-5 Well-Being Index, as well as changes in their subjective well-being through the different waves of the pandemic. Sporting habits and socio-economic variables were also surveyed, and OLS regression models were created focused on the WHO-5 measures. (3) Results: The subjective psychological well-being of the Hungarian adult population decreased significantly, but in the second and third wave of pandemic restrictions, an increase in subjective psychological well-being has been measured. The relationships between the time spent on doing sports and subjective psychological well-being were significant in each pandemic waves. The highest subjective psychological well-being and its highest increase were reported by those who could increase their time spent on doing sports as well. (4) Conclusions: The relationships between the sports activities, physical health, size of settlement, changes in income and subjective psychological well-being of the Hungarian adult population were significant in all three waves of the COVID-19 pandemic.

## 1. Introduction

The COVID-19 pandemic changed the daily life of the world’s population significantly and caused significant health-related, social, economic, and ecological consequences in almost all countries across the globe [1,2]. The wave-like spread of the pandemic claimed more than 5.2 million lives worldwide in 2020 and 2021 [3,4,5]. To contain or handle the health crisis, several countries introduced restriction measures. In the first year of the pandemic, the focus was on hygiene regulations and social distancing to slow down the spreading of the virus. From 2021, on top of these, the focus shifted to country-level vaccination coverage [6,7,8,9,10].

The negative public-health-related consequences of the necessary social distancing restrictions are researched and communicated in the relevant literature. Social isolation had a negative impact on the mental health and health-related behavior of people [11,12,13]. The opportunities for physical activities also decreased significantly due to the imposed restrictions [14,15,16].

Based on the different variants of the virus and the number of cases, three distinct waves of the pandemic have been identified at the health policy level and in the literature, as well from the outbreak to the summer 2021. The intensity of the COVID-19 pandemic had significant differences in its geographical distribution and timing across the globe [17,18,19].

This study is focused on Hungary, which was also significantly impacted by the pandemic. Over the three waves of the pandemic (from 11 March 2020 to 15 June 2021), more than 807,000 people were infected with the COVID-19 virus, which claimed around 30,000 thousand lives [18,20]. This contributed to a 15% increase in the number of deaths in Hungary compared to the estimated number of deaths without the pandemic [20,21]. It is particularly unfavorable that, as long as the number of infected people per 100,000 persons was close to the OECD countries’ average (8444.3), the number of deceased per 1,000,000 persons was the highest among developed countries (3074.4) between 2020 and October 2021 [22].

The main aim of our research was to investigate the relationship between sporting habits and subjective psychological well-being in all three waves of the pandemic on the Hungarian adult population. Therefore, we have conducted three different representative sample surveys on the subjective psychological well-being of the 18–69 year old Hungarian population during the different waves of the pandemic and analyzed their sporting habits and changes in the different periods of the restrictions. To obtain a deeper understanding, demographic, socio-cultural and economic traits of the population have been also included.

### 1.1. Subjective Psychological Well-Being and Physical Activity

The WHO-5 well-being index, introduced by the World Health Organization, is the most frequently used scale for the measurement of subjective psychological well-being [23,24,25]. This scale measures the positive aspects of subjective psychological well-being with five short questions, and it is used on both healthy and unhealthy populations [26]. The WHO-5 scale has a high level of validity and measures the intended impacts well [23,25]. With the WHO-5 scale, well-being was measured in several fields of medicine [27,28] and also in other fields, such as social capital [29] or wellbeing related research [30]. The scale is also suitable for measuring the results of clinical procedures and for comparisons with the average population or different social groups and/or periods [23,25]. The WHO-5 scale has been translated into more than 30 languages and is used in an unchanged format, worldwide [25].

According to the country comparison of the results of the European Quality of Life (EQLF) survey, the subjective psychological well-being of Hungarian people evolved favorably in the 2010s, as the results displayed a positive trend and in the last measurement before the pandemic (in 2016), Hungary was included in the top three countries in the European Union, according to the WHO-5 index [30].

According to several researchers and their results, physical activity impacts the biological, spiritual, mental, emotional, and social dimensions of health and changes in people’s quality of life in an extremely diverse and complex way [31,32,33,34,35,36]. A lack of physical activity is a risk factor, whereas regular exercise is a protective factor, in almost all non-communicable chronic diseases. According to the WHO’s estimations, a non-satisfactory quantity of physical activity could be the cause of five million annual deaths, worldwide [37]. Physical inactivity is responsible for around 6–10% of non-communicable diseases and approximately 8–9% of premature deaths [38,39]. The health-protective effect of regular physical activity predominates prevention-related areas, but it is also important in therapeutic or rehabilitation treatments. Physical activity and sport also have a therapeutic impact on mental illnesses, such as depression, anxiety, or alcohol abuse [33,40,41].

Physical activity impacts mental health in several positive ways. With physical strain, it creates a state that serves as a condition for fighting stress, and with developing physical parameters can counterbalance the negative somatic effects of mental illnesses [34,42]. Physical activities can also increase confidence, self-esteem, learning and concentration, creativity, work rate, and more relaxing sleep, all of which contribute positively to mental health even in a short time [33,34,43,44].

According to several research results, physical activity and sport have a significant positive impact on well-being [45,46,47,48,49,50,51,52]. Whilst some studies have focused on the impact of physical activity [15,47,53,54,55], others have focused on the impact of sports [2,45,46,56,57,58], but their results were in agreement. More active people will have higher well-being than physically inactive or passive people. In general, an increase in the frequency and quantity of sport or physical activity will increase well-being as well. However, Merglen [56] has shown that excessive sport will decrease well-being; therefore, the relation between well-being and sport/physical activity is rather a U-curve. The relation between well-being and the intensity of physical activity is also not linear, as higher intensity did not increase well-being more than activities with lower intensity [47,51]. Several authors argue that team activities have a more favorable impact than individual activities [49,54,56], whereas others found evidence of a more favorable impact of activities pursued in nature [51].

As most of the mentioned studies focus on physical activity, we sought to enrich the literature concerning the impact of sports, as that term is narrower than physical activity and its health and well-being related literature is also narrower.

Under the term doing sports, we mean “all forms of physical activity, which, through casual or organized participation, aim at expressing or improving physical fitness and mental well-being, forming social relationships or obtaining results in competition at all levels” [59].

In Hungary, the sporting habits or physical activity of the population increased in the 2010s as the proportion of people who exercised regular physical activity increased. Although the increment was one of the best in the European Union (EU), in absolute measures, the Hungarian population still lags from the EU average [60,61,62].

### 1.2. Sport and Subjective Psychological Well-Being in the COVID-19 Pandemic

The restrictions and lockdowns imposed because of the COVID-19 pandemic decreased the opportunities for physical activities and had a negative impact on the sports sector. Several authors have argued about the changes in physical activity, or the impact on the sports industry [63,64,65,66,67]. Some studies, which covered more continents, have concluded that, in the first two phases of the COVID-19 pandemic, the physical activity of the population decreased almost everywhere and sitting activities increased [64,68]. Studies that focused on adults experienced only a few special cases without a significant decrease in time spent on active sports [69,70,71]. The degree of the decrease in several groups was very serious and one-fifth of the studies reported, on average, a more than 50% decrease in time spent on active sports and only a few percentages of the population could increase their sporting time among the healthy adult population [64,68]. In terms of demographic and sociocultural traits, older age groups, men, and people with lower educational levels or with unfavorable economic and social status were finding fewer opportunities for physical activity and their decrease in physical activity was the highest during the pandemic [68].

Studies conducted during the COVID-19 pandemic have strengthened the evidence of the positive impact of physical activity on subjective psychological well-being in the significantly changed psychosocial and environmental conditions as well. The subjective psychological well-being of the inactive population and groups with low physical activity was, on average, more unfavorable than those with higher physical activity [15,52,66,72]. According to these studies, the decrease in physical activity impacts subjective psychological well-being negatively and it is independent of the individual’s fitness level [15]. Wendtlandt and Wicker [51] emphasized the role of nature-based and nature-neutral forms of activity, but their decrease also predicts decreasing subjective psychological well-being.

Several studies [15,73,74,75] have focused on sport and its relationship with subjective psychological well-being during the COVID-19 pandemic, although these studies were mostly cross-sectional studies focused on the first or the second wave of the pandemic. We identified the research gap in investigating the relationship between time spent on sports, as well as socio-economic background variables, on subjective psychological well-being during all three phases of the pandemic.

## 2. Materials and Methods

This research was part of the research program “Changes the Living and Working Conditions of the Hungarian Population” with a Focus on Physical Activity and Sport Consumption Habits [18]. As the COVID-19 pandemic had three official phases in Hungary, our data collection was also focused on those three phases (see Table 1). The data collection was based on the survey method with a one-on-one interview; the in-person interviews were conducted with social distancing and the participants were approached by the specialist survey company contracted to the research program, and the questionnaire was designed by the researchers. A representative set of 1200 person stratified sample was collected in each phase of the pandemic. The strata were based on sex, age, region, settlement size, and educational level and covered the 18–69 year old Hungarian population. All of our surveys were produced during the lockdown period or the given pandemic waves.

For the measurement of subjective psychological well-being, the validated WHO-5 Well-being scale has been employed. The scale was developed by Bech et al. [76] and its short version, which has 5 questions, has been selected [24]. The WHO-5 was validated for Hungary in 2002 in the Hungarostudy health survey [77] and its latest survey was organized in 2013 [78]. The Hungarian validation process changed the original 6-point scale to a simplified 4-point scale (0—not typical at all, 1—barely typical, 2—typical, 3—utterly typical), which was used in this study. Due to the scale change, the Hungarian results are measured in a 0–15 range, whereas international results are measured in a 0–25 range, but in several studies, the WHO-5 results are transmuted to a 0–100 scale for better understanding and comparison [23,30].

Sporting habits have been measured by a calculated variable from two survey items. To calculate the average time spent on sports weekly, first, we surveyed how many times a week people do sports, on average; then, we asked the average time spent on doing sports on one occasion. This is in line with the European Commission’s Eurobarometer studies [60]. The average time spent on doing sports was calculated by multiplying the two variables. 

The results from the three COVID-19 phases have been analyzed with a comparison of the descriptive statistics, the Mann-Whitney U-test and the Kruskal-Wallis test. To identify the relationships, the Pearson correlation, chi-square test and OLS regression with the enter method were used. The regression models were created with the same variables in each phase of the pandemic, where the dependent variable is WHO-5 and the main independent variable is physical activity, but it is complemented with socio-economic variables, as suggested by Babbie [79]. The socio-economic variables include age, sex, size of the settlement, education, change in income during the pandemic, physical health, and size of household (see Appendix A). All of the used variables are scale variables except sex, which is used as a dummy (0—female, 1—male). The variables were selected due to their presence in previous studies in connection with subjective psychological well-being. The requirements of the OLS regression were tested and met in each model, the multicollinearity and autocorrelation were tested in connection with the independent variables, and the homoscedasticity and normality were tested in connection with the error terms. All of the tests met the requirements; therefore, regression models are suitable.

SPSS 27.0 was used for statistical calculations and the significance level was set to 0.05.

## 3. Results

The subjective psychological well-being of the 18–69 year old Hungarian population was measured in the three phases of the COVID-19 pandemic, as can be seen in Table 2. During the lockdown of the first wave of the COVID-19 pandemic, the subjective psychological well-being of the Hungarian population decreased to 8.57 (SD = 3.14) on the Hungarian WHO-5 scale, which would mean 57.13 as a normalized value. 

Although each wave of the pandemic was worse than the one before, the subjective psychological well-being of the Hungarian adult population did not decrease accordingly. In the second wave of the pandemic, the subjective psychological well-being of the Hungarian population increased to 8.72 (SD = 2.47, 58.13 as normalized), but the difference between the two phases is not statistically significant. In the third phase of the restrictions, the subjective psychological well-being of the Hungarian population increased significantly to 9.09 (SD = 3.35, 60.59 as normalized, *p* < 0.001).

Changes in sporting habits revealed a similar trend, which can be seen in Table 3 and Table 4. In the period immediately preceding the pandemic, the sporting habits of the adult Hungarian population were more favorable than in the periods of the three waves of the pandemic.

The sporting habits of the Hungarian population were most unfavorable during the first wave of the pandemic compared to the lockdown periods of the second and, in particular, the third wave, when on average more people took part in sports. Compared to the period before COVID-19, the time spent exercising per week (from 52.69 to 28.99 min/week) and the proportion of people exercising at least weekly (from 35.8% to 21.7%) also decreased significantly (*p* < 0.001 and *p* < 0.001) by the time of the first wave. The average time spent on sports remained almost unchanged in the first and second waves, but increased significantly (*p* = 0.005) to 37.88 min/week by the time of the third wave. The proportion of regular exercisers appeared to be the lowest in the first wave, which rose to over 25.8% and 25.3% in the lockdown periods of waves two and three, respectively, but that was also far below the proportion of the pre-pandemic era.

We also examined the proportion of people who were able to maintain or even increase their sporting activity despite the pandemic and the restrictions imposed. At the time of the first wave, 12.4% of the adult Hungarian population were able to maintain their sporting habits and 1.3% took up sport or were able to increase the frequency and/or intensity of their sporting activities. In the second wave, 15.0% were able to maintain their sporting activities unchanged and only 0.1% were able to change them in a positive direction. In the period of the third wave restrictions, 15.8% of Hungarian adults were able to continue their sporting activity in the same way and 1.5% were able to change it positively compared to the period before the restrictions.

Table 5 shows the correlation between subjective psychological well-being and time spent on sports during the three waves of the restrictions imposed because of the COVD-19 pandemic. There is a significant, although weak, correlation between time spent on sports and the WHO-5 well-being measures in each wave of the pandemic, which means those people who spent more time on sports reported higher well-being.

Table 5 also shows that those who exercised at least weekly during the restriction periods had significantly higher WHO-5 scores than those who did not exercise or exercised only occasionally in all three of the periods studied. For the group of regular athletes, their WHO-5 scores show an upward trend between waves, in contrast to the trend for those who do not or rarely spend time doing sport.

When focusing on behavior changes during the restrictions, significant differences were also observed in the time spent on sports and subjective psychological well-being relations. Those who were able to maintain or improve their sporting behavior (e.g., taking up the sport or increasing time spent on doing sports) reported significantly more positive subjective psychological well-being in all three waves of the pandemic than the groups of non-athletes and those who did fewer sports compared to the pre-restriction period. In each wave, those who could improve the time spent on doing sports had the highest WHO-5 scores of all the sport-related groups studied and showed a significant increase in their subjective psychological well-being index score between waves (significance of Kruskal-Wallis tests for waves 1 and 2 *p* = 0.045, waves 2 and 3 *p* = 0.008). It is important to highlight that those whose sporting behavior changed negatively during the period of restriction had similar (not significantly different on average) WHO-5 scores to the non-athletes’ group, regardless of their time spent on doing sports.

### Relationship of Sports and Subjective Psychological Well-Being

The regression models on the subjective psychological well-being during the different waves of the pandemic can be seen in Table 6. All three models were significant and met all of the required criteria.

The variables included in the model explained 23.3% of the variation in the subjective psychological well-being during the period of the first wave of restrictions and six factors had a significant impact in the model. These were, in order of the strength of impact on physical health (ß = 0.392): size of settlement (ß = 0.231), change in income (ß = 0.163), education (ß = 0.106), time spent on doing sports (ß = 0.057) and sex (ß = 0.052).

During the second wave of the pandemic, the same eight variables included in the model explained 29.8% of the variation in WHO-5. Similar to the first wave, the independent effect of the six explanatory variables remained significant in this model. In the lockdown period of the second wave, physical health and settlement size were the most strongly influencing factors, along with the continued significant influences of change in income, education, time spent on doing sports, and age. Sex and household size were not independently influential in the second wave period.

The explanatory power of the model for the third wave was 31.2%, which is the highest of our multivariate regression models. After controlling for the interacting effects of the explanatory variables, the impact of four variables remained significant on WHO-5. In this period, subjective psychological well-being was most strongly influenced by physical health, with change in income, size of the settlement, and time spent on doing sports being other explanatory factors with independent effects.

To summarize the three results from the three models, physical health, size of the settlement, change in income, and time spent on doing sports were significantly associated with subjective psychological well-being in all three periods of the pandemic.

## 4. Discussion

The latest pre-pandemic studies on subjective psychological well-being in Hungary show that the normalized WHO-5 score of the Hungarian adult population was 64.18 in 2013 [78] and 69 in 2016 [30], establishing the Hungarian adult population one of the highest scoring populations in the EU. Hungary, similarly to other European countries, has been severely affected by the COVID-19 pandemic, with significant consequences in many areas of life, which have also had a negative impact on the mental health of the population. Mental health was particularly affected by the periods of restriction that formed the time frame of our study [18,22]. Our results show a significant decrease in the WHO-5 scores (*p* < 0.001 between each wave and the Hungarostudy results), particularly in the first and second waves of the pandemic. In the third pandemic wave, we measured a significantly more favourable subjective psychological well-being rate than in the period of the first two waves; however, this was still below the pre-pandemic conditions (see Table 7).

The “U” shaped correlation caused by the improved outcomes of the third wave may be conditionally influenced by the increase in knowledge about the nature of the epidemic and its management, as well as the advent of the vaccination and the associated population-based vaccination programme [18]. The improvement in subjective psychological well-being from the trough of the first wave is consistent with the expectations of the “set point” theory, the main idea of which is that the well-being of adults is stable because it is determined by personality traits and genetic or partly genetic factors. The inherited or learned factors develop the set point, which does not change or changes only temporary (because of significant life-changing events, such as the birth of a child, a family tragedy, or even the impacts of a global pandemic); however, in time the individual’s subjective psychological well-being returns to the set point [80,81]. Our results are similar to those found in studies that used the set point theory to support the claim that a pandemic as a negative life event adversely affects psychological well-being, but that the majority of individuals tend to return to their natural set point after a period of time [82,83,84]. EQLF surveys were conducted by Eurofound during different periods of the pandemic; therefore, their comparisons with our results would be problematic [85].

The emergence of the COVID-19 pandemic has severely affected the sports sector. In addition to the changes in professional sports, the conditions and framework for recreational and health-protecting sports have also changed significantly. The restrictive measures implemented during the COVID-19 pandemic have had a negative impact on people’s sporting habits. During the lockdown periods, the forms and locations of physical activity also changed significantly. The forms of sport that can be practiced alone in nature or in the home have become more valued [51,68,86,87]. The results of our research clearly show that the pandemic and the associated restrictive measures also led to very significant changes in the sporting habits of the Hungarian adult population. National and international surveys conducted before the pandemic measured the proportion of the Hungarian adult population participating in sports at least weekly to be between 33–39% [60,88]. This means that the Hungarian adult population were below the average EU participation rates in sports in all of the measurement periods. Our survey shows that 35.8% of Hungarian adults participated in sports at least weekly in the period immediately preceding the pandemic, which is in line with the results from previous years.

During the period of the first restrictions, introduced at the end of spring 2020, there was a significant drop of almost 40% in the proportion of people taking part in regular sport. A similar drop occurred in the closely related average time spent on doing sports, with a drop of almost 45% by the time of the first lockdown. The period of the first lockdown also saw the most unfavorable conditions in terms of participation in sport, as shown by the lowest proportion of people (13.7%) who were able to either maintain their sporting activity or change their sporting activity in a positive direction. In the period of restrictions linked to the second wave, there were already positive changes in some parameters (e.g., the proportion of people who exercise regularly), but the average time spent on doing sports is at a similarly low level (29 min/week) as in the first wave. In contrast, there is a significant improvement in the lockdown period of the third wave, where the time spent on doing sports (37.9 min/week) and the proportion of people who at least maintained their sporting habits (17.3%) are significantly higher than in the first wave. Despite the improved trend, the measured parameters show a significant lag in the third wave compared with pre-pandemic levels. The positive change between the waves is presumably facilitated by the sports sector’s pandemic management efforts, the innovations introduced (e.g., the development of online training formats and technologies [86,89,90], and the stabilization of changing sporting habits [64,68]. The magnitude and trends of changes to the time spent on doing sports in the Hungarian adult population follow a similar pattern as seen in many other developed countries [64].

According to the positive association between the time spent on sports and subjective psychological well-being during the COVID-19 pandemic, our results are in line with several other studies [14,15,51,52,72,73,74,75]. However, in this study, this association was also found in all three waves of the pandemic. Faulkner et al. [15] found that, according to combined data from the United Kingdom, New Zealand, Ireland and Australia in the first wave of the COVID-19 pandemic-induced restrictions, there was a moderate positive correlation between Physical Activity and the WHO-5 scores of individuals (Spearman rho = 0.35, *p* < 0.001). Similarly, Wendtlandt and Wicker [51] found that during the first wave of the COVID-19 pandemic, the time spent on nature-based sport activities was positively related to subjective psychological well-being (*p* < 0.01), although they did not disclose the correlation coefficients.

However, it is interesting to see that, in our research, the effect size was quite small in the first wave of the pandemic (Hedges g = 0.29 between those subjective psychological well-being who could increase time spent on sports or did not change and those who decreased time spent on sports) and could only increase to medium in the second (g = 0.58) and third (g = 0.6) wave.

We also emphasize those reported the highest WHO-5 scores who could either maintain or increase their time spent on sports during the restrictions of the pandemic, even if it was lower than the amount recommended by the WHO. This is in line with Faulkner et al.’s [15] multi-country cross-sectional study, however, our findings also show that members of this group could significantly increase their subjective psychological well-being in each wave of the pandemic, which was not the case with those who did not engage with sport or who could only decrease the time spent on sports.

Based on previous research, the variables included in the multivariate regression models could be assumed to relate to subjective psychological well-being. Each regression model demonstrated a significant relationship between the independent variables and subjective psychological well-being. In the restriction periods of the pandemic, the strongest influencing factor was physical health. The association between physical health and mental or subjective psychological well-being has been reported in several studies [91,92,93,94,95,96,97,98,99]. The results obtained in our study suggest that subjectively perceived poorer physical health is associated to poorer subjective psychological well-being among the Hungarian adult population in each wave of the COVID-19 pandemic. The role of physical health—particularly the lack of it—as associated with well-being is confirmed by several studies conducted in the pandemic periods [9,100,101,102]. It can be understood, however, that the presence of disease was of particular importance to the mental well-being of people in periods when a significant part of the country’s health services were not available, or were only available to a very limited extent [103,104].

In addition to physical health and time spent doing sports, the independent variables of settlement size and changes in income were significantly associated with subjective psychological well-being in all three models. The model suggests that, in Hungary, the subjective psychological well-being of residents who reside in larger settlements are greater than those who reside in smaller settlements; this is in line with previous Hungarian research, which associates this phenomenon with the limited labour market and quality of life opportunities of smaller settlements [105,106,107]. The differences observed are strongly determined by the social, economic, and settlement geography characteristics of the given country [101,108,109,110,111,112,113,114,115,116].

Studies conducted before and during the pandemic have also provided extensive evidence that income and changes in income have a significant influence on mental well-being. The subjective psychological well-being of those with lower or decreasing income is worse than that of those with high or increasing income [30,51,72,115,117,118,119]. This relationship can also be seen in our study, as changes in income was among the three most significantly associated items in each model.

The role of the other variables included in this study were not stable throughout the pandemic waves in relation to the WHO-5 measure; where they were significant in some waves of the pandemic, they were not significant in other waves. According to sex, men and women’s subjective psychological well-being were different before and during the pandemic. The pandemic period clearly accentuated the differences [22,30,72]; however, in our study, sex was only significant in the first period. The association between a higher level of education and more favourable WHO-5 scores was confirmed by several studies both during and before the pandemic [30,72,120]; this was also the case for the Hungarian adult population in the first and the second wave of the pandemic, but somehow education lost its significant association with subjective psychological well-being in the third wave. Both variables have been confirmed in previous research to influence the WHO-5 scores. According to the influence of age on subjective psychological well-being, studies show that older adults had more favourable WHO-5 scores than younger adults during the pandemic [72,101]. In our study, age was only significant in the second wave of the pandemic and the WHO-5 values of younger age groups were more favourable, which deteriorated with age. We suggest that this is because, in the second wave, younger generations had more awareness about the pandemic due to their higher accessibility and usage of the internet, but we did not have any survey items on this; therefore, this is just a suggestion which could be an interesting future research direction.

## 5. Conclusions

In this study, we explored the relationship of time spent on doing sports and several socio-economic variables with the subjective psychological well-being of the Hungarian adult population during the three waves of the COVID-19 pandemic. The time spent on doing sports was significantly and positively associated with subjective psychological well-being of the Hungarian adult population, but its effect size was rather small in the first wave of the pandemic and only medium in the second and third waves. Between waves, the highest subjective psychological well-being and the highest increase in subjective psychological well-being were reported by those who could increase their time spent on doing sports.

An interesting future research direction could be how the subjective psychological well-being of the Hungarian adult population would change if another crisis were to occur, which seems to be the case as another socio-economic crisis is developing.

The limitation of our study is that we did not have direct data on the subjective psychological well-being of the Hungarian adult population immediately before the COVID-19 pandemic; to tackle this limitation we used all of the available data. Different regression models with different influential variable models could have been created for each pandemic wave, but we decided to use similar models to investigate the influence of the identical set of variables during each pandemic wave to compare those associations. Using more socio-economic items could have increased the explanatory power of our models, but the profile of our survey limited the scope of the socio-economic questions and the explanatory power of the models happened to be good as well.

According to our results, policymakers should focus on further educating the population regarding the association between sports and well-being and trying to create further opportunities for recreational sports.

## Figures and Tables

**Table 1:** Data collection.

Timing	Sample Collection	Sample Size	Method
1st wave	25–30 May 2020	1200 person	survey method with a one-on-one interview
2nd wave	7–13 December 2020	1200 person	survey method with a one-on-one interview
3rd wave	30 April–6 May 2021	1200 person	survey method with a one-on-one interview

Source: Authors’ edition.

**Table 2:** Subjective psychological well-being of Hungarian population (18–69 years).

	n	Mean (0–15)	Std. Deviation	Mean (0–100)	Kruskal-Wallis Test *p* Value	
					2nd Wave	3rd Wave
1st wave	1200	8.57	3.14	57.14	0.607	<0.001 ***
2nd wave	1200	8.72	2.47	58.09		<0.001 ***
3rd wave	1200	9.09	3.11	60.59		

*** significance level *p* < 0.01. Source: Authors’ calculation from research database

**Table 3:** Changes in sporting habits during the different waves of the COVID-19 pandemic.

	n	Time Spent on Sports (Min)	Std. Deviation	Proportion of People Sporting Weekly (Regular)	Not Changed or Increased Time Spent on Sports
				*p* < 0.001	*p* = 0.045
Before the 1. wave	1200	52.69	95.79	35.8%	
1st wave	1200	28.99	77.86	21.7%	13.7%
2nd wave	1200	29.01	60.53	25.8%	15.1%
3rd wave	1200	37.88	89.64	25.3%	17.3%

Source: Authors’ calculation from the research database.

**Table 4:** Significance levels of the differences of sporting habits under the different waves of the pandemic.

Significance Levels from Wave to Wave	Kruskal-Wallis Test *p* Value	
	2nd Wave	3rd Wave
1st wave	0.995	0.005 *
2nd wave		0.005 *

* significance level *p* < 0.01. Source: Authors’ calculation from the research database.

**Table 5:** Correlations of subjective psychological well-being and time spent doing sport.

	1st Wave		2nd Wave		3rd Wave	
**Time Spent on Sport—WHO-5** (Pearson Correlation)	**Pearson** **Correlation**	**Sig.** **(2 taled)**	**Pearson** **Correlation**	**Sig.** **(2 taled)**	**Pearson** **Correlation**	**Sig.** **(2 taled)**
average time spent on doing sports weekly (min)	0.133 **	<0.001	0.198 **	<0.001	0.180 **	<0.001
**Regular sport—WHO-5** (Kruskal-Wallis Test)	**mean (SD)**	**Sig.** **(2 taled)**	**mean (SD)**	**Sig.** **(2 taled)**	**mean (SD)**	**Sig.** **(2 taled)**
doing sports regularly	9.15 (3.07)	<0.001	9.67 (1.96)	<0.001	9.95 (2.91)	<0.001
rarely or not doing sport at all	8.42 (3.15)		8.38 (2.55)		8.80 (3.12)	
**Changes in sporting habits—WHO-5** (Kruskal-Wallis Test)	**mean (SD)**	**Sig.** **(2 taled)**	**mean (SD)**	**Sig.** **(2 taled)**	**mean (SD)**	**Sig.** **(2 taled)**
time spent on sports increased or did not change *	9.29 (3.36)	1 = 0.003 2 = 0.010 3 = 0.663	9.87 (1.99)	1 < 0.001 2 < 0.001 3 = 0.354	10.59 (2.45)	1 < 0.001 2 < 0.001 3 = 0.812
did not spent time on sport **	8.48 (3.11)		8.49 (2.57)		8.78 (3.12)	
decreased time on sports ***	8.36 (2.99)		8.68 (2.10)		8.85 (3.26)	

significance levels 1 means between * and **, 2 between * and ***, 3 between ** and ***. Bold is to highlight the name of the columns and variables. Source: Authors’ calculation from the research database.

**Table 6:** Regression models on WHO-5 in the different waves of the pandemic.

	1st Wave			2nd Wave			3rd Wave		
	B	ß	Sig	B	ß	Sig	B	ß	Sig
(Constant)	2.240		<0.001	3.945		<0.001	2.777		<0.001
Age	0.001	0.006	0.829	−0.011	−0.068	0.017	0.007	0.034	0.201
Sex	0.324	0.052	0.043	0.092	0.019	0.450	−0.020	−0.003	0.896
Size of household	0.086	0.034	0.203	−0.044	−0.022	0.409	−0.127	−0.048	0.053
Physical health	1.368	0.392	<0.001	0.987	0.396	<0.001	1.687	0.517	<0.001
Education	0.130	0.106	<0.001	0.108	0.117	<0.001	0.001	0.001	0.976
Size of settlement	9.613 × 10^−7^	0.231	<0.001	1.151 × 10^−6^	0.352	<0.001	6.025 × 10^−7^	0.147	<0.001
Change in income	1.144 × 10^−5^	0.163	<0.001	1.277 × 10^−5^	0.133	<0.001	1.301 × 10^−5^	0.183	<0.001
Time spent on doing sports (min/week)	0.002	0.057	0.036	0.003	0.069	0.007	0.004	0.109	< 0.001
	R^2^ = 0.233			R^2^ = 0.298			R^2^ = 0.312		
	F = 46.257			F = 63.974			F = 68.636		
	Sig < 0.001			Sig < 0.001			Sig < 0.001		
	Durbin-Watson = 2.012			Durbin-Watson = 1.749			Durbin-Watson = 1.898		
	VIF = 1.007–1.168			VIF = 1.016–1.375			VIF = 1.012–1.226		

Source: Authors’ calculation from the research database.

**Table 7:** Subjective psychological well-being of the Hungarian population in the different surveys.

Survey	WHO-5 Wellbeing Index (Mean 0–100)
EQLF (2007)	63.00
EQLF (2011)	61.00
Hungarostudy (2013)	64.18
EQLF (2016)	69.00
1st wave (May 2020)	57.14
2nd wave (Dec 2020)	58.09
3rd wave (May 2021)	60.59

Source: Authors’ calculation from research database, Susánszky and Székely [78] and Eurofound [30].

## Data Availability

The dataset supporting the conclusions of this article is available from the corresponding author on reasonable request.

## Appendix A

Items of WHO-5 scale

Over the last two weeks …

1. I have felt cheerful and in good spirits
2. I have felt calmed and relaxed
3. I have felt active and vigorous
4. I woke up feeling fresh and rested
5. My daily life has been filled with things that interest me

**Table A1:** Independent variables of the regression models.

Variables	Question	Attributes
Age	How old are you?	… year old
Sex	What is your gender?	0—female, 1—male
Size of household	How many people live in your household?	… person
Physical health	How would you rate your current physical health?	Likert scale 1–5 1—the worst possible … 5—the best as possible
Education	Number of years of successful schooling?	… year
Size of settlement	Number of inhabitants of your municipality?	… person
Change in income	Has your income situation changed during the pandemic?	… Forint
Time spent on doing sports (min/week)	How often do you exercise or play sport? * In general, on days when you do exercise or play sport, how much time do you spend at it?	… min/week
